# Chromium (VI) Inhibition of Low pH Bioleaching of Limonitic Nickel-Cobalt Ore

**DOI:** 10.3389/fmicb.2021.802991

**Published:** 2022-01-11

**Authors:** Ana Laura Santos, Agnieszka Dybowska, Paul F. Schofield, Richard J. Herrington, Giannantonio Cibin, D. Barrie Johnson

**Affiliations:** ^1^School of Natural Sciences, Bangor University, Bangor, United Kingdom; ^2^Natural History Museum, London, United Kingdom; ^3^Diamond Light Source, Didcot, United Kingdom; ^4^Health and Life Sciences, Coventry University, Coventry, United Kingdom

**Keywords:** acidophiles, Cr (VI) toxicity, limonite, nickel-cobalt laterites, reductive bioleaching, XANES spectroscopy

## Abstract

Limonitic layers of the regolith, which are often stockpiled as waste materials at laterite mines, commonly contain significant concentrations of valuable base metals, such as nickel, cobalt, and manganese. There is currently considerable demand for these transition metals, and this is projected to continue to increase (alongside their commodity values) during the next few decades, due in the most part to their use in battery and renewable technologies. Limonite bioprocessing is an emerging technology that often uses acidophilic prokaryotes to catalyse the oxidation of zero-valent sulphur coupled to the reduction of Fe (III) and Mn (IV) minerals, resulting in the release of target metals. Chromium-bearing minerals, such as chromite, where the metal is present as Cr (III), are widespread in laterite deposits. However, there are also reports that the more oxidised and more biotoxic form of this metal [Cr (VI)] may be present in some limonites, formed by the oxidation of Cr (III) by manganese (IV) oxides. Bioleaching experiments carried out in laboratory-scale reactors using limonites from a laterite mine in New Caledonia found that solid densities of ∼10% w/v resulted in complete inhibition of iron reduction by acidophiles, which is a critical reaction in the reductive dissolution process. Further investigations found this to be due to the release of Cr (VI) in the acidic liquors. X-ray absorption near edge structure (XANES) spectroscopy analysis of the limonites used found that between 3.1 and 8.0% of the total chromium in the three limonite samples used in experiments was present in the raw materials as Cr (VI). Microbial inhibition due to Cr (VI) could be eliminated either by adding limonite incrementally or by the addition of ferrous iron, which reduces Cr (VI) to less toxic Cr (III), resulting in rates of extraction of cobalt (the main target metal in the experiments) of >90%.

## Introduction

Lateritic deposits account for over 70% of accessible nickel reserves (Dalvi et al., 2004) and can also contain significant amounts of cobalt and copper (Ñancucheo et al., 2014; Santos et al., 2020). These are stratified oxidised regoliths, with most of the metals of commercial value found in magnesium silicate-rich saprolite zones that underly iron oxy-hydroxide-rich limonitic zones. While saprolite is mined as a nickel ore, limonite is often not processed and is stockpiled as a waste material. While technologies exist that enable the extraction and recovery of base metals from limonites [such as the Caron or high-pressure acid leach (HPAL) processes] these can be highly demanding of energy and have significant carbon footprints (Stanković et al., 2020). In contrast, bioprocessing laterites using autotrophic acidophilic bacteria has been demonstrated to effectively solubilise nickel, cobalt, and copper at ambient temperatures and atmospheric pressure with less consumption of acid than other (non-biological) processing strategies (e.g., Ñancucheo et al., 2014; Marrero et al., 2015). This process uses bacteria that couple the oxidation of zero-valent (elemental) sulphur (ZVS) to the reduction of iron (III) and manganese (IV) at low pH (<2), thereby accelerating the dissolution of minerals such as goethite and asbolane. Since most of the nickel and cobalt are associated with oxidised minerals in limonite deposits, their destruction releases target transition metals and these are retained in solution in the acidic liquors, facilitating their downstream recovery. As this approach (the reduction of oxidised minerals) is essentially the reverse of that used in conventional bioprocessing operations, it has been referred to as “biomining in reverse gear” (Johnson and du Plessis, 2015).

Most published studies of limonite bioprocessing have described laboratory-scale experiments, though there has been at least one pilot-scale test at a laterite mine in northern Brazil (D. B. Johnson et al., 2013). Many of the experiments described have been carried out using stirred bioreactors in which pH, temperature, etc. can be maintained and monitored, and with solid densities of 1–5% (w/v) (e.g., Marrero et al., 2015; Santos et al., 2020) which are well below those that would be used in a commercial operation (typically 20% for stirred tank mineral bioprocessing operations). While it has been found that most, if not all, limonite deposits so far tested appear to be amenable to reductive bioprocessing (though with some degree of variation; Santos et al., 2020), investigating how effective this is when operating under conditions that better reflect commercial constraints is an area that requires more in-depth study.

The microorganisms used in reductive mineral bioprocessing are mostly sulphur-oxidising bacteria of the genera *Acidithiobacillus* and *Sulfobacillus* that can also catalyse the dissimilatory reduction of ferric iron and have been widely documented to display greatly enhanced tolerance (compared to most life forms) to a wide range of cationic transition metals including iron, nickel, and cobalt (e.g., Norris and Ingledew, 1992). However, they are also highly sensitive to other transition metals, such as molybdenum, vanadium, and chromium, when these are present as oxy-anions or undissociated acids (Dopson et al., 2014). For example, Johnson et al. (2017) found that *Acidithiobacillus* and *Leptospirillum* spp. grew in laboratory media containing 10–100 mM cationic chromium (III) but were totally inhibited by much smaller concentrations (<5 μM) of chromium (VI), which is mostly present as anionic hydrogen chromate, HCrO_4_^–^, at pH 1–2. While chromium (VI) would not be anticipated to be present in reduced (sulphidic) ores, it, and other oxy-anionic metals, have the potential to be present in oxidised ore deposits and therefore to inhibit bioleaching.

During the course of testing the amenability of limonites from New Caledonia to reductive bioprocessing at 10% solids density, it was found that not only was bioleaching ineffective but also that the bacteria appeared to be killed in leach liquors. The objective of this study was to find the reason for this inhibition and to devise a method to circumvent it.

## Materials and Methods

### Origin of the Limonite Samples Used in Experimental Work

Two samples of lateritic limonite (NC1 and NC2) were obtained from the Penamax mine in New Caledonia (Maurizot et al., 2020). Both had been ground and screened to <100 μm particle diameter. In one experiment, limonite NC2 that had been screened to <50 μm (NC2_<50_) was used.

### Mineralogical and Elemental Analysis

Mineralogical and chemical analyses of limonite samples were carried out using a combination of techniques, described below. Bulk chemical analyses were performed using induction coupled plasma mass spectrometry (ICP-MS) and induction coupled plasma atomic emission spectroscopy (ICP-AES) as described by Johnson et al. (2020). Samples were prepared by lithium metaborate/lithium tetraborate fusion for major elements (Si, Fe, Al, Ca, K, Mn, Ti, Cr, Na) by 4-acid digestion for trace elements and analysed at ALS Global Laboratories (Loughrea, Ireland). X-ray powder diffraction (XRD) patterns were collected with a PANalytical X’Pert Pro α1 MPD diffractometer equipped with an Xcelerator real-time strip-detector that has an active detector length of 2.122° and using Co Kα radiation. Data were recorded in continuous mode over 4–90 degrees 2⊖ with 0.0170 step size and a scan rate of 0.01°s^–1^, resulting in total scan times of ∼2 h. The powders were packed into an aluminium sample deep well and phase identifications performed by pattern matching using the Powder Diffraction File (PDF) database of the International Centre for Diffraction Data (ICDD) and standard material from the mineral collection at the Natural History Museum (United Kingdom).

Electron microprobe analysis (EMPA) was performed using wavelength-dispersive X-ray spectrometry (WDX) on a Cameca SX100 electron microprobe, operating at 20 kV accelerating voltage, 20 nA current and a 1 μm spot size. All data were matrix-corrected using the Cameca version of the PAP PhiRhoZ programme (Pouchou and Pichoir, 1984a,b). Quantitative SEM-EDX was performed using the Oxford Instruments INCA XMax Energy Dispersive Spectrometer (EDS) on the Zeiss EVO 15LS scanning electron microscope (SEM). Objective lens to specimen working distance was kept constant at 10 mm (fixed focus). The electron beam accelerating voltage was 20 kV, and electron beam current 1.5 nA. Quant optimisation was performed on cobalt metal, typically every 3 h. The accuracy of EDX analysis was checked regularly at each session by collecting spectra and quantifying elemental concentrations in the reference sample of Kakanui augite (Jarosevich et al., 1980). Beam current was regularly checked throughout the analysis session. Point spectra and large area EDX maps were collected on the carbon coated polished block samples. Data were processed using the Oxford Instruments AzTech software.

X-ray absorption near edge structure (XANES) spectroscopy was performed using beamline B18 at Diamond Light Source, United Kingdom (Dent et al., 2009). The energy was selected using a Si(111) monochromator; Pt coated Si mirrors were used to collimate and focus the X-ray beam; high energy harmonics contributions from the Si(333) reflection were removed by inserting in the beam path a parallel pair of Pt coated Si mirrors at 8 mrad grazing incidence angle. Data were collected in fluorescence mode with pressed pellets of powdered samples mounted vertically in reflection geometry and the XPRESS3 Si-drift fluorescence detector set at 90° to the incident beam. The distance between the detector and the sample was adjusted to ensure that the total incoming count rate was within the linear range of the detector. Spectra were acquired in continuous scanning (Qexafs) mode from 5796 to 6795.5 eV recording 3999 points in a scan time of 240 s with 9 spectra recorded for each sample and merged. The energy was calibrated by setting the position of the first maximum of the first derivative of the XANES spectrum of a Cr foil to 5989 eV. Spectra were background subtracted and normalised using Athena (Ravel and Newville, 2005) and the pre-edge features extracted by fitting the resulting background to a spline function. The pre-edge features were fitted using Gaussian components.

### Bioleaching and Abiotic Leaching of New Caledonian Limonite

#### Microbial Cultures

Consortia of acidophilic sulphur-oxidising, iron-reducing prokaryotes were used in bioleaching tests. The mesophilic consortium included *Acidithiobacillus* (*At.*) *ferrooxidans^T^* and “*At. ferruginosus^T^*,” *At. ferriphilus^T^*, *At. ferridurans^T^*, *Sulfobacillus* (*Sb.*) *thermosulfidooxidans^T^*, and the moderate thermophilic consortium contained *Acidianus* (*Ac.*) *brierleyi^T^*, *Ac. sulfidivorans^T^*, and *At. caldus* strain BRGM3 (Moya-Beltrán et al., 2021). Cultures were sourced from the *Acidophile Culture Collection* maintained at Bangor University except for the *Acidianus* species which were obtained from the *German Collection of Microorganisms and Cell Cultures* (DSMZ, Germany). A starter culture of each consortium (each inoculated with ∼10^8^ cells of each species) was set up in shake flasks containing basal salts and trace elements (Ñancucheo et al., 2016), 100 μM ferrous sulphate and 5% (w/v) zero-valent sulphur (ZVS; supplied by VWR Chemicals United Kingdom, and sterilised at 110°C for 1 h). The same liquid medium amended with 0.02% (w/v) yeast extract was used for the moderate thermophilic consortium. For the mesophilic consortium, the liquid medium pH was set initially at 2.5 and flasks were incubated at 35°C, and for the moderate thermophilic consortium the liquid medium was initially pH 1.5 and cultures incubated at 48°C.

#### Bioleaching Set-Up

Four sequential bioleaching experiments were carried out using New Caledonian NC1 or NC2 limonite (Table 1) in 2 L (working volume) reactor vessels coupled to FerMac 310/60 units (Electrolab Biotech, United Kingdom) that controlled pH (by automated addition of 1 M sulphuric acid), temperature, and agitation (Santos et al., 2020). In experiment (I), NC1 limonite was added (at 10%, w/v) to a bioreactor, maintained at 35°C and pH 1.5, containing the mesophilic consortium. Experiment (II) used the same operational parameters as those in experiment (I), except that NC1 limonite was added incrementally (Table 1), to give a final solids density of 15%. Experiment (III) used NC2 limonite and the moderate thermophilic consortium, with the bioreactor maintained at pH 1.3 and 50°C. Ferrous sulphate was added (to 2 mmoles L^–1^) to the bioreactor ahead of the limonite, which was again added incrementally to a final solid density of 10%. Experiment (IV) also used the moderate thermophilic consortium and NC2 limonite, with the bioreactor maintained at pH 1.5 and 50°C. Ferrous sulphate was added (to 5 mmoles L^–1^) ahead of the first addition of NC2 limonite, and further amounts of NC2 were added to the reactor incrementally, reaching a final total solid density of 15% w/v (Table 1). Experiment (V) investigated abiotic leaching of NC2 limonite at pH 1.5 and 50°C and 15% solids density using the same FerMac 310/60 control units and 2 L reactor vessels but with 1 L working volume. Ferrous sulphate (10 mmoles L^–1^) was added to the limonite suspension on three separate occasions over the 4-day experiment (Table 1).

**TABLE 1:** Conditions and operational parameters of the (bio) leaching experiments carried out with NC1 and NC2 limonite.

Exp.	Limonite sample	Microbial consortium	pH/T (°C)	Fe(II) addition (mmoles L^–1^)	Limonite addition (%, w/v)
(I)	NC1	Meso*	1.5/35	-	10 (day 0)
(II)	NC1	Meso*	1.5/35	-	1 (day 0); 4 (day 15); 5 (day 29); 5 (day 43)
(III)	NC2	MT**	1.3/50	2 (day 0)	1 (day 0); 4 (day 7); 5 (day 14)
(IV)	NC2	MT**	1.5/50	5 (day 0)	0.5 (day 2); 1 (day 3); 1.5 (day 4); 2 (day 7); 2.5 (day 8); 2.5 (day 9); 2.5 (day 11); 2.5 (day 14)
(V)	NC2	None (abiotic)	1.5/50	10 (0 h) 10 (21 h) 10 (44 h)	15 (day 0)

*Mesophilic consortium; **Moderately thermophilic consortium.

In all experiments, the limonite suspensions were stirred at 150 rpm and sparged with oxygen-free nitrogen (OFN) to remove dissolved oxygen. Volumes of acid added to maintain set *pH* values were recorded and cumulated. Liquid samples were withdrawn from the reactor vessels at regular intervals to measure redox potentials [using a platinum/silver-silver chloride electrode (Thermo Scientific, United Kingdom) and adjusted to be relative to a standard hydrogen electrode, i.e., *E*_*H*_ values], concentrations of ferrous iron (using the Ferrozine colorimetric assay; Stookey, 1970), total iron (by reducing ferric iron to ferrous and repeating the Ferrozine assay) and other selected transition metals (manganese, cobalt, nickel, and chromium) by AAS (using a SpectrAA Duo atomic absorption spectrophotometer; Varian, United Kingdom). At the end of each experiment [except experiment (III)], solid and liquid phases were separated by filtration through Whatman (United Kingdom) #1 filter papers. Leachates were stored at 4°C, and solid residues dried at room temperature, ground to fine powders using a pestle and mortar, and their mineralogical compositions analysed.

### Comparison of Concentrations of Chromium (VI) and Other Soluble Metals in NC2_<50_ Limonite Leached Abiotically With Acidic Solutions Containing Either Magnesium Sulphate or Ferrous Sulphate, and Assessment of the Biotoxicities of These Leachates

Finer grain size (NC2_<50_) limonite was abiotically leached (20%, w/v) using solutions of either 100 mM ferrous sulphate or 100 mM magnesium sulphate, both adjusted to pH 0.5 with sulphuric acid. Leaching was carried out in sealed bottles, shaken at 100 rpm at 48°C for 2 days, after which the liquid and solid phases were separated by centrifugation (4,000 × *g*, 5 min). Concentrations of chromium (VI) in the leachates were determined colorimetrically using diphenylcarbazide (Pflaum and Howick, 1956), and other metals by ICP-OES (Agilent 5800) with analysis in axial mode and calibrated with ICP multi-element standards (Sigma-Aldrich, United Kingdom).

An empirical assessment of the comparative toxicities of the leachates obtained was determined by adding different aliquots of them to a liquid medium containing 10 mM ferrous iron, basal salts and trace elements (Ñancucheo et al., 2016), inoculating with a consortium of iron-oxidising acidophiles (a mixture of *Acidithiobacillus* and *Leptospirillum* spp.), incubating shake flasks at 30°C for up to 10 days and monitoring ferrous iron oxidation.

## Results

### Elemental and Mineralogical Analysis of NC1 and NC2 Limonite

Comparative elemental analysis of New Caledonian limonite samples, including the finer fraction NC2_<50_, are shown in Table 2. Limonite NC1 was rich in Si, and Fe, and also contained appreciable amounts of Mg, Ca, and Al, but relatively low concentrations of Mn. In contrast, NC2 contained much smaller amounts of Si, Ca, and Mg, but again large amounts of Fe. The chromium content of NC2 was over twice that of NC1, and NC2 also contained far higher concentrations of the two target metals (in terms of their commercial value), cobalt and nickel. Some subtle differences were apparent between NC2 screened to <50 μm to that screened to <100 μm, most notably in the finer fraction (NC2_<50_) containing less cobalt and manganese, though both contained similar amounts of nickel.

**TABLE 2:** Elemental compositions of the New Caledonian limonite samples.

	NC1	NC2	NC2_<50_		NC1	NC2	NC2_<50_
Fe	231	487	499	Ni	3.0	13.5	13.7
Si	141	10	11	Co	0.42	1.6	0.53
Al	47	29	26	Zn	0.18	nd	0.41
Ca	24	0.07	0.50	Cu	0.06	0.06	0.05
Mg	50	0.28	1.14	Ti	1.42	0.12	0.12
Na	1.0	0.28	0.30	V	0.37	0.16	Nd
K	0.08	0.083	0.083	Sc	0.12	0.06	Nd
Cr	8.3	20	14.2	LOI*	11.6	16.0	15.2
Mn	4.5	9.2	5.2				

*LOI: loss on ignition, %.

*All values shown as g kg^–1^, unless otherwise stated; nd, not determined.*

**FIGURE 1:**
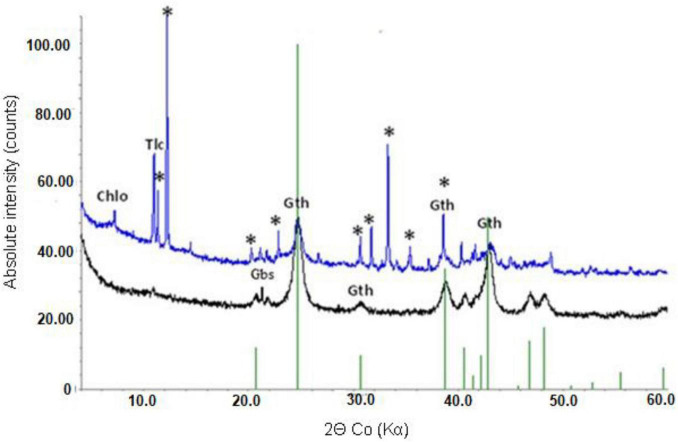
X-ray powder diffraction patterns of NC1 (top) and NC2 (bottom). Chlo-chlorite, Gth-goethite, Tlc-talc, Gbs-gibbsite, *-hornblende.

Mineralogical analysis supported chemical analysis of the limonite samples. XRD analysis of NC1 limonite identified the following crystalline phases: hornblende, talc, goethite, chlorite, haematite, smectite, glauconite, and magnetite (Figure 1). While the manganese concentration in this sample was relatively low and no Mn oxide/oxy-hydroxide minerals were identified with XRD, Mn minerals (mainly asbolane-lithiophorite intermediates), along with chromite, were identified using SEM-EDX. No silicate or carbonate phases were detected in NC2 using XRD, reflecting its low Si, Ca, and Mg contents. Its XRD pattern was almost identical to that of a pure goethite standard, which was the main crystalline phase detected (Figure 1). In addition, a small amount of gibbsite was detected by XRD. The manganese content of NC2 limonite (9.2 g kg^–1^) was about twice that of NC1 (4.5 g kg^–1^), and again while no Mn-oxide/oxy-hydroxide minerals were identified with XRD, they were (along with chromite) identified using SEM-EDX. The XRD pattern of NC2_<50_ was very similar to that of NC2.

The mineralogy of Co-rich grains in sample NC1 was investigated using an analytical SEM Zeiss EVO. Distinct and liberated Mn and Co rich grains were identified. SEM-EDX analysis revealed Co to Ni ratios varying from 0.2 to 1.5 with Co concentrations averaging 5.7 wt% (1.1–9.6 wt%) and Ni 7.3 wt% (1.9–11.4 wt%), Fe 7.8 wt%, (2.8–13 wt%) Mn 24.3 wt% (18–31 wt%), and Al 5.1 wt% (1.8–9.8 wt%), with no other elements detected in substantial quantities (above 0.5 wt%). Based on their chemical composition these grains were classified as asbolane-lithiophorite intermediates.

SEM-EDX analysis of NC2 limonite found that Fe-oxide grains were ubiquitous, as expected from chemical and XRD analysis. SEM imaging revealed this phase to be very porous while Quantitative EMPA combined with SEM-EDX analysis (analytical totals, Fe content and trace element chemistries) indicated that Fe-oxides were mostly goethite with some haematite present (as supported by XRD analyses). Fe-oxides contained Si, Al, and small quantities of Ni and Cr, though Co was not detected in this phase using SEM-EDX (detection limit 0.3–0.4 wt%). In addition, small chromite grains were also identified with SEM and found to be distributed throughout the sample. Manganese-rich phases were observed as either very small inclusions in goethite grains or as very fine veins within the porous goethite matrix. Two types of Mn oxy-hydroxides were identified in NC2 limonite, a hydrated cryptomelane (a K-bearing hollandite group Mn-oxide) and asbolane-lithiophorite intermediate. The Mn oxy-hydroxides were quantitatively analysed to measure the Co content, and for cryptomelane this was found to vary from 0.13 to 0.34% with an average of 0.27 wt%. Other elements detected in this phase were K (average of 3 wt%), Fe (average of 7.3 wt%) and Al, Ni, Na, Ba (all below 0.5 wt%). The asbolane-lithiophorite intermediates were found to have almost equal concentrations of Co (average of 7.4 wt% and ranging 2.8–9.8 wt%) and Ni (average of 8.1 wt% and ranging 3.7–11 wt%). Other elements detected in these phases were Al (average of 3.4 wt% and ranging 2.4–4.1 wt%), Fe (average 9.1 wt% and ranging 4.4–18.8 wt%), Na and Mg (<0.5 wt%).

### Bioleaching New Caledonian Limonites

**FIGURE 2:**
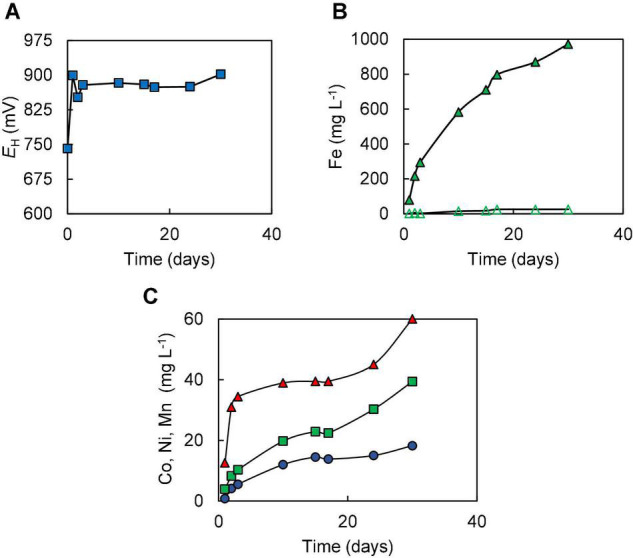
Changes in **(A)** redox potentials, **(B)** concentrations of ferrous iron (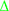) and total iron (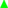), and **(C)** concentration of metals [cobalt (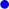); nickel (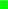); manganese (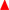)] during the bioleaching of NC1 limonite added at 10% (w/v) solids density at time 0 (experiment I).

In Experiment (I) where bioleaching of 10% w/v NC1 limonite added at the start of the experiment was tested, it was noted that redox potentials increased from ∼ +750 mV to over +900 mV very soon after addition of the ore and that similar redox potential values (+875–+902 mV) were maintained throughout the 30-day experiment (Figure 2A). *pH* values also increased quickly after adding limonite (from 1.5 to 1.8) but returned to the pre-set value as sulphuric acid was pumped into the reactor; ∼140 mmoles of sulphuric acid were consumed in maintaining the pH of the reactor over the course of experiment (I). Concentrations of soluble Fe (II) were consistently very low (∼28 mg L^–1^) whereas those of Fe (III) increased during the experiment, reaching ∼950 mg L^–1^ by day 30 (Figure 2B). Analysis of soluble metals showed limited solubilisation of iron (4%), manganese (13%), chromium (4%), and nickel (12%) over the course of this experiment, though more extensive dissolution of cobalt (41%) (Figure 2C). Qualitative phase contrast microscopic examinations of mineral suspensions taken from the reactor showed that cell numbers decreased dramatically during the first few days of experiment, and no cells were observed on day 30.

**FIGURE 3:**
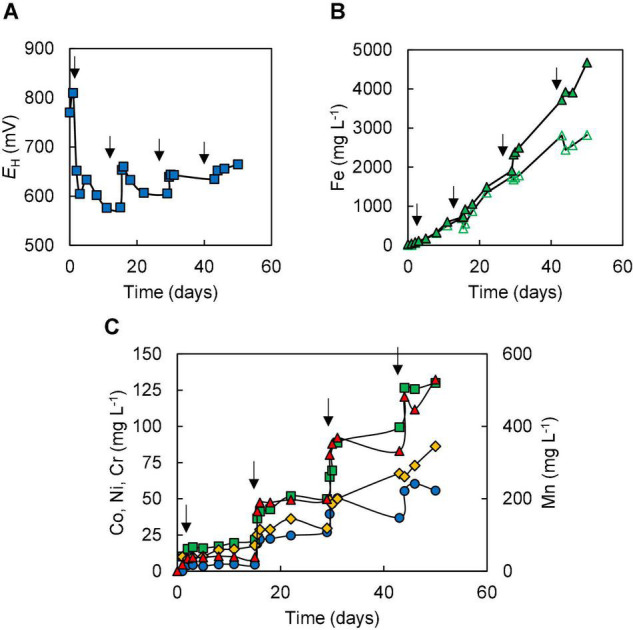
Changes in **(A)** redox potentials, **(B)** concentrations of ferrous iron (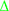) and total iron (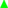), and **(C)** concentrations of metals [cobalt (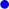); nickel (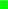); chromium (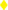); manganese (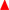)] during bioleaching of NC1 limonite added incrementally to a final solids density of 15% w/v (experiment II). Downward-pointing arrows indicate times at which NC1 limonite was added to the reactor vessel.

In contrast to data recorded in experiment (I), incremental addition of NC1 limonite to the reactor vessel in experiment (II) resulted in the development of reducing conditions, evidenced by redox potentials declining to +630–+650 mV shortly after each addition of the ore (Figure 3A) and the presence of large concentrations of ferrous iron. Concentrations of total soluble iron gradually increased during the experiment, reaching ∼4,700 mg L^–1^ by day 50, of which 70–90% was present as Fe (II) (Figure 3B). Incremental addition of limonite also resulted in far greater solubilisation of both cobalt (94%) and manganese (86%) than in experiment (I) (Figure 3C), and dissolution of both nickel (31%) and iron (15%) were also marginally greater than in experiment (I). About 8% of the total amount of chromium in NC1 limonite (∼86 mg L^–1^) was leached during the course of the experiment. About 4-times more sulphuric acid (∼566 mmoles) was required to maintain the bioreactor pH at 1.5 in experiment (II) than in experiment (I). Microscopic examination of mineral suspensions showed that cell numbers began to decrease from day 29 and, again, no cells were observed at the end of this experiment.

**FIGURE 4:**
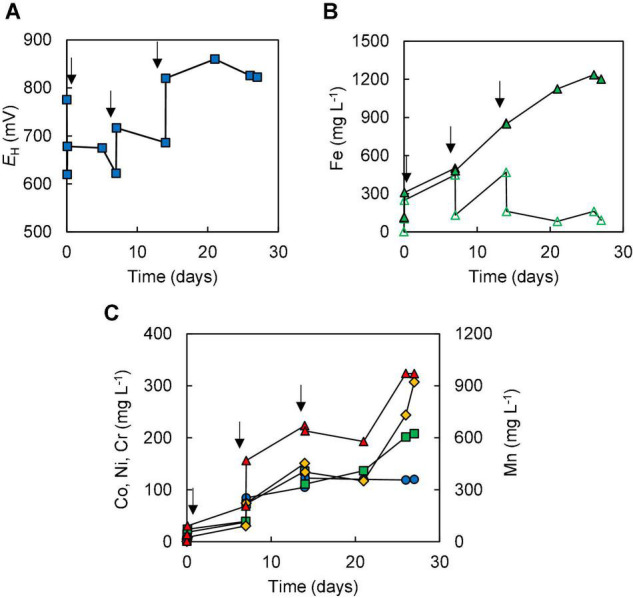
Changes in **(A)** redox potentials, **(B)** concentrations of ferrous iron (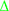) and total iron (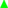), and **(C)** concentrations of metals [cobalt (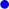); nickel (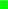); chromium (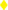); manganese (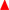)] during bioleaching of NC2 limonite added incrementally to a reactor vessel to a final solids density of 10% w/v (experiment III). Downward pointing arrows indicate times at which NC2 limonite was added to the reactor vessel.

In the two bioleaching experiments (III and IV) carried out with NC2, ferrous sulphate was added before limonite, which was added incrementally. In experiment (III), the addition of ferrous sulphate caused the redox potential of the sulphur suspension to decline from +780 to +620 mV, but this increased (to +678 mV) following the addition of 1% (w/v) NC2 limonite. After 7 days, the redox potential had fallen to ∼ +620 mV corresponding to >90% of soluble iron being present as Fe (II) (Figure 4), and more NC2 was added to the reactor (to 5% w/v, in total). This caused the redox potential to increase to > +700 mV, and this remained at ∼ +690 mV, corresponding to ∼55% of total soluble iron present as Fe (II), over the next 7 days. Adding a further 5% (w/v) of NC2 limonite (5%, w/v) at day 14 caused the redox potential to increase to >+800 mV, and the mineral leachate remained at similar relatively high values for the duration of this experiment. Again, solubilisation of cobalt (73%) and manganese (63%) exceeded that of nickel (15%) and iron (2.5%) (Figure 4C). Concentrations of soluble chromium reached 310 mg L^–1^, corresponding to about 15% of that present in NC2 limonite being solubilised in this experiment. Far less sulphuric acid (∼180 mmoles) was consumed in this experiment than in experiment (II). Cell numbers declined dramatically after the third addition of NC2 limonite (10% w/v solids density, in total) and no cells were observed in the mineral leachate from day 26 onwards.

**FIGURE 5:**
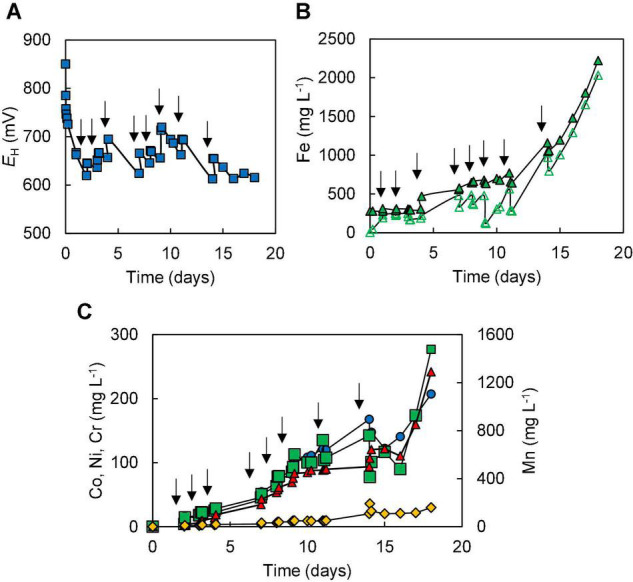
Changes in **(A)** redox potentials, **(B)** concentrations of ferrous iron (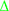) and total iron (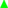), and **(C)** concentrations of metals [cobalt (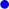); nickel (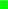); chromium (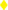); manganese (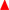)] during bioleaching of NC2 limonite, added incrementally to the reactor vessel to a final solids density of 15% w/v (experiment IV). Downward pointing arrows indicate times at which NC2 limonite was added to the reactor vessel.

The amount of ferrous iron added initially to the reactor was increased to 5 mmoles L^–1^ in experiment (IV), in an attempt to offset further a potential inhibition of microbial activity due to chromium (VI), as discussed below [Fe (II) reduces Cr (VI) to Cr (III)]. In this experiment, redox potentials initially followed a similar trend to those in experiment (III), decreasing from +850 to +620 mV after adding ferrous sulphate then increasing to +645 mV after the first addition of limonite (to 0.5% w/v). As in experiment (III), redox potentials become more positive following further additions of NC2 limonite and subsequently declined [due to microbial reduction of Fe (III)], stabilising at < +650 mV (Figure 5).

Concentrations of most soluble metals analysed were higher in experiment (IV) than in experiment (III) due at least in part to the greater final solids’ density used (Figure 5C), though in terms of percentage extraction these were similar in both experiments [72% Co, 80% Mn, 12% Ni, and ∼ 3% Fe in experiment (IV)]. One notable exception was chromium, where concentrations in experiment (IV) peaked at 160 mg L^–1^, corresponding to net solubilisation of 4.5% of this metal, both values being far below those found in experiment (III). Significantly more sulphuric acid (280 mmoles) was consumed in experiment (IV) than in experiment (III) (180 mmoles). Microscopic examinations showed that the number of cells again gradually declined with time, though in contrast to experiment (III), a small number of cells were still observed in mineral leachates after 18 days. In addition, it was noted that coccoid archaeal cells were far more abundant than bacterial rods.

### Abiotic Leaching of New Caledonian NC2 Limonite

**FIGURE 6:**
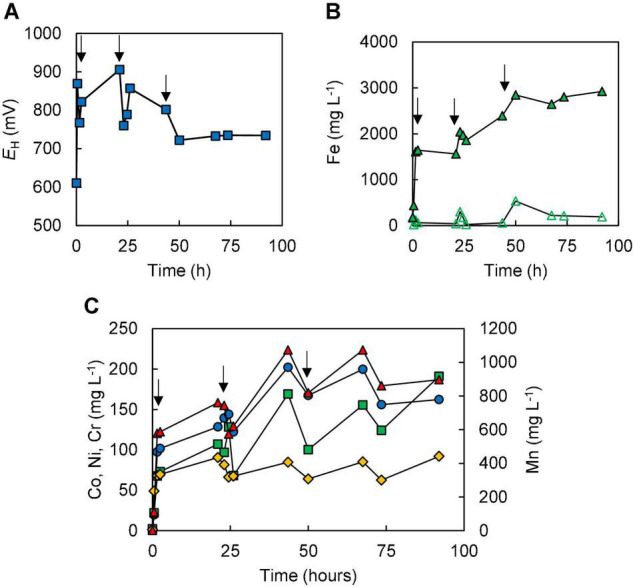
Changes in **(A)** redox potentials, **(B)** concentrations of ferrous iron (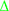) and total iron (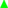), and **(C)** concentration of metals [cobalt (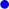); nickel (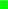); chromium (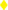); manganese (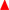)] during abiotic ferrous iron leaching of NC2 limonite at pH 1.5 and 15% (w/v) solids density [experiment (V)]. Downward pointing arrows indicate the three occasions when ferrous sulphate was added to the reactor (to 10 mmoles L^–1^).

Experiment (V) acted both as an abiotic control and a means to determine how much ferrous iron was required to react with the more readily reducible minerals [chiefly manganese (IV) minerals] and (potentially) chromium (VI). As shown in Figure 6A, each addition of ferrous sulphate caused the redox potential of the limonite suspension to fall, though this subsequently increased as the ferrous iron was oxidised to ferric iron. All of the ferrous iron added on the first two occasions was completely oxidised, though there was some (∼3.4 mmoles L^–1^) remaining 48 h after the third addition of ferrous iron (Figure 6B). The amount of ferrous iron consumed on reacting with NC2 limonite at pH 1.5 was equivalent to ∼177 mmoles kg^–1^ of limonite. Concentrations of metals (Co, Ni, Mn, and Cr) rapidly increased after the addition of NC2 limonite (15% w/v solids density) to the reactor vessel containing 10 mmoles L^–1^ ferrous sulphate (Figure 6C). Subsequent additions of ferrous sulphate (at 21 and 44 h) caused short-term falls in metals concentrations, though these increased again with time. In contrast, most of the chromium extracted from NC2 was solubilised by acid within the first 2 h of the experiment and appeared to be little affected by later additions of ferrous iron. During the course of experiment (V), ∼70% of both cobalt and manganese were extracted from NC2 limonite, and smaller amounts of nickel (10%) and chromium (3%). The mass balance of iron, taking into account that added as ferrous sulphate, indicated that ∼ 3% of the iron in the limonite sample had been solubilised. Table 3 summarises the percentage of metals extracted in each of the five experiments.

**TABLE 3:** Percentage of metals extracted in bioleaching experiments (I)–(V) and the abiotic leaching experiment (V).

	Co	Ni	Mn	Cr	Fe
I	41	12	13	4	4
II	94	31	86	8	15
III	73	15	63	15	2.5
IV	72	12	80	4.5	3
V	70	10	70	3	3

### Mineralogical Analysis of the (Bio)Leached Limonite Residues

**FIGURE 7:**
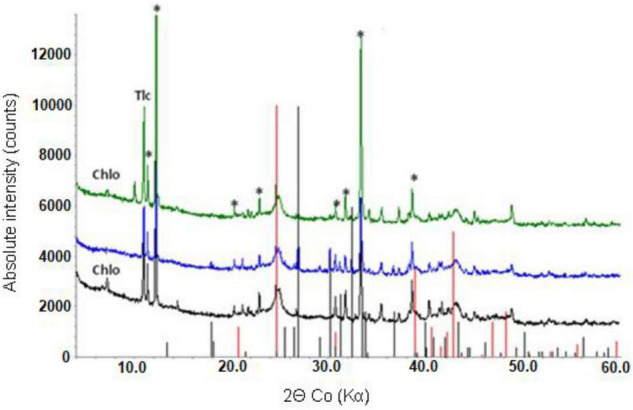
X-ray powder diffraction pattern of raw NC1 limonite (bottom), and bioleached NC1 residues from experiment (I) (middle) and experiment (II) (top). Key: Chlo-chlorite, Gth-goethite, Tlc-talc, *-hornblende. Peak positions marked for goethite (red) and sulphur (black).

**FIGURE 8:**
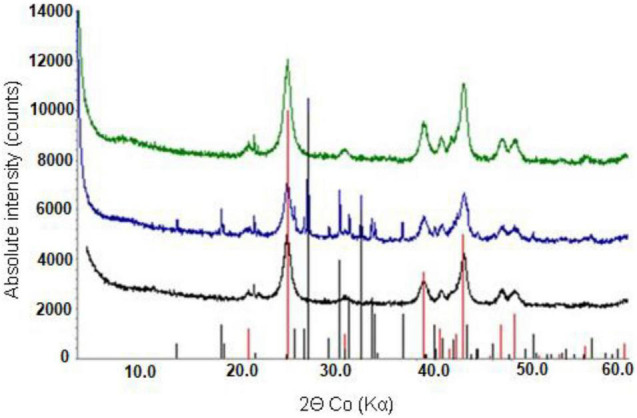
X-ray powder diffraction patterns of raw NC2 limonite (bottom), and (bio) leached NC2 residues from experiment (IV) (middle) and experiment (V) (top). Peak positions marked for goethite (red) and elemental sulphur (black).

X-ray powder diffraction patterns of NC1 residues showed that the chlorite peak intensity was either greatly reduced or no longer detected after bioleaching this limonite (Figure 7). In contrast, the peak intensity ratios for goethite, hornblende and talc indicated that bioleaching had caused no major changes to these phases. No major differences were observed in the XRD patterns of the raw and bioleached NC2 limonite (Figure 8). Elemental sulphur, which was added to provide the electron donor for the bioleaching acidophiles was identified in residues of both NC1 and NC2 limonite.

**FIGURE 9:**
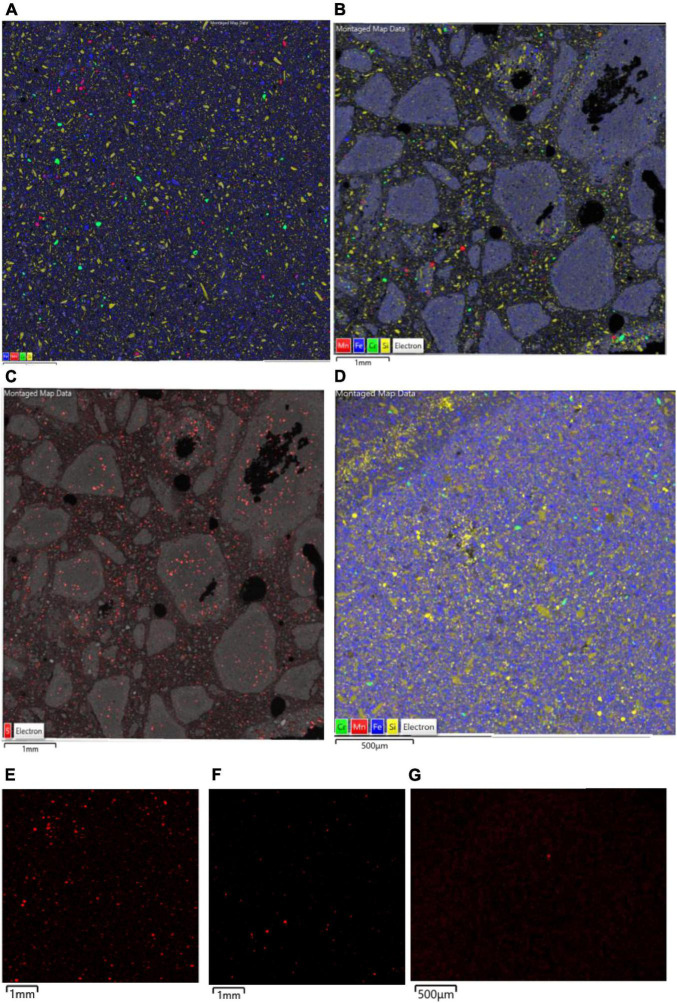
SEM-EDX maps of **(A)** raw, and **(B–D)** bioleached NC1 limonite, indicating the distribution of iron (blue, representing Fe oxy-hydroxides), chromium (green, representing chromite), manganese (red, representing Mn oxy-hydroxides), and silicon (yellow, representing silicates) overlaid on the backscattered electron image (BSE) of the mapped area. Images **(B,C)** are residue samples from experiment (I) [with image **(C)** illustrating the distribution of sulphur in red] and image **(D)** is from experiment (II). The lower images **(E,G)** are X-ray elemental maps of manganese in each sample: **(E)** raw NC1, **(F)** residue from experiment (I), **(G)** residue from experiment (II).

**FIGURE 10:**
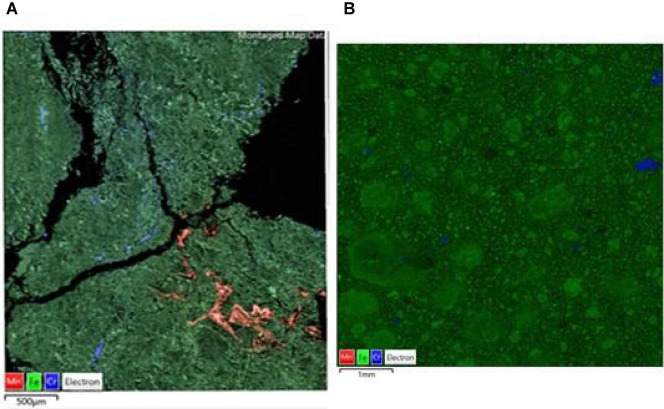
SEM-EDX maps of **(A)** raw, and **(B)** bioleached [experiment (IV) NC2 limonite, indicating the distribution of iron (green, representing Fe oxy-hydroxides), chromium (blue, representing chromite), manganese (red, representing Mn oxy-hydroxides) overlaid on the BSE image of the mapped area.

Comparison of raw and bioleached NC1 and NC2 limonite from SEM analysis (Figures 9, 10) showed that iron and chromium were abundant in both (representing Fe oxy-hydroxides and chromite) but that there was a significant reduction in abundance of Mn-rich grains in the bioleached residues, supporting chemical analysis of the leachate liquors.

### Chromium Deportment and Speciation in NC1 and NC2 Limonites

The total chromium content of NC2 limonite (2%) was greater than that of NC1 (0.83%) (Table 2) and, although no primary Cr minerals were identified with XRD, chromite [were the metal is present as Cr (III)] was identified in both samples using SEM-EDX. Chromium was also found as a trace element in Fe oxides in both limonites as well as in hornblende in NC1 limonite. The mean (and range) values of total chromium detected in NC1 were 0.25 wt% (0.03–0.91%) in hornblende, 2.83 wt% (0.14–4.75%) in haematite, and 1.17 wt% (0.02–4.75%) in goethite grains. In NC2 limonite no silicates were identified, and all of the chromium was distributed between chromite and Fe oxides. The Fe oxides as a group can be rationalised as goethite with an average of 0.21 wt% (in the range 0.03–1.38 wt%) and haematite with an average of 0.18 wt% Cr (in the range 0.03–1.93%).

**FIGURE 11:**
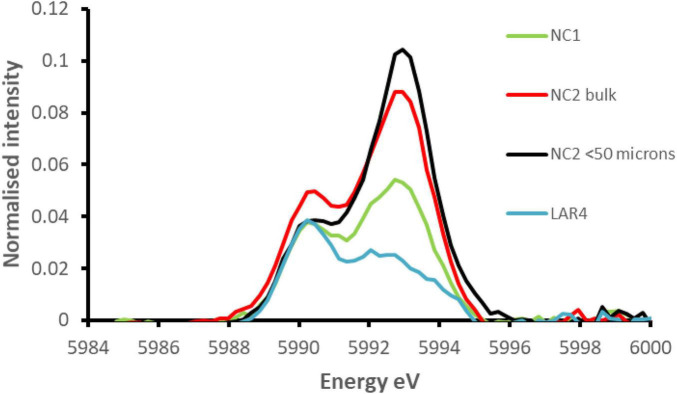
Comparison of the extracted pre-edge features of the normalised XANES spectra for NC1 and NC2 limonites from Penamax (New Caledonia) and a limonite sample from Ag Ioannis Mine (LAR4), Greece (described by Santos et al., 2020).

Figure 11 shows the pre-edge region of the Cr K-edge XANES spectra for NC1 and NC2 limonite with that from a limonite sample from the Ag Ioannis Mine (LAR4), Greece (Santos et al., 2020). XANES analysis revealed that these features comprised two distinct components, with maxima at ∼5990 eV and ∼5993 eV. The normalised intensity of the peak at ∼5993 eV is known to be an excellent indicator of the presence of chromium (VI) (e.g., Peterson et al., 1997; Huggins et al., 1999). The relative intensities of these two peaks vary, to a small degree, depending on the deportment of chromium (III) between chromite, goethite, and haematite, with the latter two producing pre-edge features comprising two peaks of equal intensity (Fandeur et al., 2009). The pre-edge feature for the Greek limonite resembled closely that of chromite, with a small contribution from chromium (III) in Fe-oxides, whereas the peak intensity at 5993 eV was seen to be significantly greater in the New Caledonian limonites, especially NC2 and NC2_<50_ samples. This is strongly indicative of the presence of chromium (VI) in both NC1 and NC2 limonites (Fandeur et al., 2009). Using beamline specific methods similar to those described by Peterson et al. (1997) and Fandeur et al. (2009), the proportion of chromium that is present as Cr (VI) in NC1, NC2, and NC2_<50_ was estimated to be 3.1% (±0.04), 6.6% (±0.1), and 8.0% (±0.2), respectively.

### Speciation of Chromium in Acid Leachates of NC2_<50_ Limonite, and Toxicities of Leachate to Iron-Oxidising Acidophilic Bacteria

Table 4 compares the chemistries of liquors obtained by leaching NC2_<50_ limonite with dilute sulphuric acid (pH 0.5) containing 100 mM of either magnesium sulphate or ferrous sulphate. While about half of the soluble iron present in the ferrous sulphate leach liquor had been oxidised to ferric (discounting the ferrous iron that would have arisen from the acid dissolution of ferrous iron-containing minerals), the redox potential of this leachate was far less positive than that of the acidic magnesium sulphate leachate, where >99% of the soluble iron was present as iron (III). The higher pH of the ferrous iron leachate can be accounted for by ferrous iron reduction of manganese hydrous oxides, which is an acid-consuming reaction. Much more manganese was leached from the limonite using the acidic ferrous sulphate solution, as were also cobalt and nickel, both of which are associated with Mn oxy-hydroxides. Whereas concentrations of total soluble chromium were greater in the acidic ferrous sulphate extracts, chromium (VI) was not detectable, in contrast to the acidic magnesium sulphate extract.

**TABLE 4:** Comparison of leachate chemistries obtained by treating 20% (w/v) NC2_<50_ limonite with pH 0.5 sulphuric acid solutions containing 100 mM magnesium sulphate or ferrous sulphate.

	MgSO_4_	FeSO_4_		MgSO_4_	FeSO_4_
pH	0.84	0.94	Mn	1.32	11.6
*E*_*H*_ (mV)	+920	+685	Co	0.18	1.83
Fe_*total*_	40.0	131	Ni	0.57	2.38
Fe (II)	0.34	58.4	Al	27.8	30.7
Cr_*total*_	1.41	2.63	Ca	1.37	1.33
Cr (VI)	0.23	<0.01	V	nd	0.02

*All metal concentrations are in mmoles L^–1^; nd, not detected.*

The two leachates were very different in their apparent toxicities to iron-oxidising acidophiles. Ferrous iron oxidation occurred in cultures containing the magnesium suphate leachate diluted 10-fold (or more) but not in those where it was diluted fivefold. In contrast, no bacterial iron oxidation was observed in cultures containing the ferrous iron leachate diluted 50-fold or less, though it did occur when it was diluted 100-fold.

## Discussion

Conventional biomining operations involve using acidophilic microorganisms to accelerate the oxidative dissolution of sulphide minerals, thereby either solubilising target metals such as copper (bioleaching) or allowing associated metals such as gold to be accessed by chemical lixiviants (bio-oxidation), and are mostly carried out in dumps, aerated heaps, and stirred tanks (Johnson, 2014). In contrast, oxidised ores require microorganisms to reduce metals (chiefly iron) to accelerate the reductive dissolution of minerals such as goethite and asbolane and uses acidophilic populations that couple the oxidation of sulphur to the reduction of ferric iron allows the metals released to remain in solution, facilitating their downstream recovery. Reductive mineral dissolution is favoured by anoxic conditions, though aerobic pure cultures of some sulphur-oxidising acidophiles can also promote the reduction of ferric iron (Marrero et al., 2015; Johnson et al., 2021).

Limonitic laterite samples from diverse global locations have been found to be amenable to reductive bio-processing, though most reported laboratory tests have been carried out in stirred bioreactors at relatively low (2.5–5% w/v) solids density (e.g., Hallberg et al., 2011; Ñancucheo et al., 2014; Smith et al., 2017; Santos et al., 2020). In the current study, NC1 limonite added *en bloc* at 10% (w/v) completely inhibited the microbial reduction of iron, allowing only acid dissolution to occur (Figure 2). The fact that incremental addition of NC1 limonite facilitated reductive dissolution to occur as evidenced both by lower *E*_*H*_ values of leachates (Figure 3A) and far greater consumption of acid (reductive dissolution of goethite and Mn oxy-hydroxides are proton-consuming reactions) suggested that a component of this limonite was acid-leached to an inhibitory concentration at 10% w/v but not at smaller solids densities, and that the ferrous iron generated by sulphur-oxidising bacteria in the absence of oxygen removed the inhibiting reagent to at least some degree. The latter hypothesis was supported by bioleaching experiments carried out with NC2 limonite, where adding ferrous sulphate to the bioreactor ahead of the limonite promoted reductive bio-processing, more so when added at 5 mmoles L^–1^ (Figure 5) than at 2 mmoles L^–1^ (Figure 4). Chromium (VI) was considered as a possible primary agent responsible for the observed inhibition of microbial iron reduction in the bioreactors. This exists mostly in the form of soluble, oxy-anionic HCrO_4_^–^ in low pH, high redox potential liquors, and is extremely toxic to acidophiles (Johnson et al., 2017). Ferrous iron is well known to reduce chromium (VI) to far less biotoxic chromium (III), and this appears to be why this form of iron, generated either biologically or added independently circumvented the problem of Cr (VI) inhibition of reductive bio-processing of these Penamax limonites. The data suggested that it is critical that chemical chromium (VI) reduction proceeds rapidly, in order not to expose the bioleaching bacteria to this oxy-anion, which causes their rapid inactivation.

The presence of chromium (VI) in limonites from New Caledonia was previously reported by Fandeur et al. (2009) who found that up to 25% of total chromium in a limonite deposit (Koniambo) situated 27 m below the land surface was present as chromium (VI) and that this value reached 33% in some localised points. They postulated that this arose within the limonite from the oxidation of chromium (III) by Mn oxy-hydroxides and suggested that the chromium (III) was released from the Fe-oxides located at Mn-oxyhydroxide boundaries and oxidised to chromium (VI) before being adsorbed to the surfaces of the surrounding Fe-oxides. There is no evidence that acidophilic (or other) microorganisms are able to oxidise chromium (III) to chromium (VI) (Johnson et al., 2017), and there was no evidence of chromite dissolution in the current (bio)leaching experiments. This suggests that, in the natural environment, the localised dissolution of Cr-bearing Fe-oxides followed by oxidation of Cr (III) and subsequent adsorption of chromate on a mineral (Fe-oxide) surface may well be the source of the chromium (VI) present in the raw Penamax limonites. Also noteworthy is that NC2_<50_ limonite which would have had a higher mineral surface area than the <100 μm bulk NC2 material had a noticeably higher proportion of chromium (VI), even though it had a smaller total Cr content. The analytical data that showed that NC2 limonite contained about twice the amount of Mn oxy-hydroxides (Table 2) and substantially more chromium (VI) than NC1 limonite (Figure 11) also support the hypothesis of Fandeur et al. (2009).

Additional evidence of the presence of chromium (VI) in NC2 limonite came from analysis of acid extracts that contained either magnesium sulphate or ferrous sulphate. The redox potential of the former was 235 mV more positive than the latter, reflecting primarily differences in iron concentrations and speciation in the two extracts (Table 4). While the published standard *E*_0_′ of the Cr (VI)/Cr (III) couple is +1.33 V, the measured *E*_0_′ in acidic sulphate-rich liquors has been reported to be somewhat less positive, at ∼ +0.840–+0.895 V (Johnson et al., 2017). This implies that chromium (VI) would have been stable in the high redox potential acidic magnesium sulphate extracts, which was found to be the case, though chromium (VI) concentrations in the far lower *E*_*H*_ acidic ferrous sulphate extracts were below detection limits (Table 4).

Incremental addition of NC1 limonite to the bioreactor and development of a relatively low redox potential leachate liquor (Figure 3) allowed far more cobalt and manganese, and also more nickel and iron, to be solubilised than when a single addition (at 10%, w/v) was made. The reductive dissolution of Mn oxy-hydroxides, and release of associated cobalt and nickel, would have been catalysed by the ferrous iron generated by the acidophilic microbial consortium, and the dis-equilibrium caused in iron chemistry would have induced more rapid acid dissolution of the goethite phase which would have also contributed to soluble nickel in the leachates (Hallberg et al., 2011). In addition, ferrous iron released from dissolution of acid-labile minerals such as chlorite would have also contributed to the reductive dissolution of Mn oxy-hydroxides, and presumably also to the reduction of some of the soluble Cr (VI) released by acid dissolution.

Ferrous iron was effective at reducing Cr (VI) and thereby removing the inhibition of limonite bioleaching caused by HCrO_4_^–^. However, and somewhat paradoxically, the leachate liquors generated by leaching NC2_<50_ limonite with an acidic lixiviant containing 100 mM ferrous sulphate was far more inhibitory to microbial iron oxidation than a control solution that contained 100 mM magnesium sulphate. The reason for this was not apparent. The osmotic potentials of the two extracts were similar, as were concentrations of many of the soluble metals analysed (with the notable exceptions of manganese, cobalt and nickel), but all of which were (individually) well below the upper levels tolerated by iron-oxidising acidophiles (e.g., Norris et al., 2020). It is possible that combinations of elevated concentrations of two of more of these metals were responsible for the observed microbial toxicity, though the fact that between 50- and 100-fold dilution of the ferrous sulphate extracts was required to obviate its inhibition of iron oxidation suggests that the agent(s) responsible were acutely toxic and not (as was HCrO_4_^–^) transformed to less inhibitory forms by reaction with ferrous iron. Research into this unexpected finding is continuing.

The potential impact of this work in the development of reductive bio-processing of oxidised ores, such as Penamax limonite, that contain relatively large amounts of highly toxic oxy-anions (hydrogen chromate in the current instance) that are readily extracted by mineral acid, is highly significant. The key reaction in the process is microbial ferric iron reduction, and if this is retarded or completely inhibited [as in experiment (I)], metals are solubilised from limonite by acid dissolution alone, which is less effective (e.g., Hallberg et al., 2011). Ferrous iron reduces Cr (VI) to less toxic Cr (III) and it is pertinent that the first stage in a published biohydrometallurgical concept for processing limonitic laterites (the Ferredox process; du Plessis et al., 2011) involves recirculating acidic ferrous iron-rich liquor into the reactor where the limonite is reduced, which would eliminate the chromium hazard. However, the possibility that acidic ferrous iron lixiviants can produce liquors that are themselves highly biotoxic has implications for the choice of techniques used for downstream processing.

## Conclusion

Reductive bioleaching of limonitic laterites may be inhibited by Cr (VI), released by dissolution of this oxy-anion in acidic liquors. The inhibition can be partially or completely eliminated by either adding ferrous iron to reduce Cr (VI) to less toxic Cr (III), or by incremental addition of limonite, whereby ferrous iron produced by microbial reduction of ferric iron released by acid dissolution again catalyses the reduction of Cr (VI). Acidic ferrous iron extracts of limonites can also be highly biotoxic due to factors that are currently not known.

## Data Availability Statement

The original contributions presented in the study are included in the article/supplementary material, further inquiries can be directed to the corresponding author.

## Author Contributions

AS: experimental and analytical work (bioleaching), and preparing figures and tables. AD: chemical and mineralogical analysis, and preparing figures and tables. PS: chemical and mineralogical analysis, XANES data analysis, and preparing figures and tables. RH: conceptual contributions. GC: XANES experiments. DJ: experimental work (abiotic leaching) and writing manuscript. All authors contributed to editing the manuscript and approved the submitted version.

## Conflict of Interest

The authors declare that the research was conducted in the absence of any commercial or financial relationships that could be construed as a potential conflict of interest.

## Publisher’s Note

All claims expressed in this article are solely those of the authors and do not necessarily represent those of their affiliated organizations, or those of the publisher, the editors and the reviewers. Any product that may be evaluated in this article, or claim that may be made by its manufacturer, is not guaranteed or endorsed by the publisher.
